# Iris tomate

**DOI:** 10.11604/pamj.2014.17.240.4236

**Published:** 2014-03-29

**Authors:** Omar Lezrek, Rajae Daoudi

**Affiliations:** 1 Université Mohammed V Souissi, Service d'Ophtalmologie A de l'hôpital des spécialités, Centre Hospitalier Universitaire, Rabat, Maroc

**Keywords:** Iris tomate, synéchies, glaucome, Iris tomato, synechiae, glaucoma

## Image en medicine

L'iris tomate ou iris bombé est une condition où il y a une apposition de l'iris au cristallin ou au vitré antérieur (par des synéchies sur 360°), empêchant l'humeur aqueuse de s’écouler de la chambre postérieure à la chambre antérieure; la pression dans la chambre postérieure augmente et entraînant une courbure antérieure de l'iris périphériques et l'obstruction du réseau trabéculaire. Il peut en résulter une crise aiguë de glaucome à angle fermé. Il s'agit d'une patiente de 42 ans, suivie pour uvéite totale bilatérale avec mal observance thérapeutique qui consulte pour oeil douloureux gauche, avec à l'examen une acuité visuelle à PL+, un TO à 48 mmHg avec un aspect d'iris tomate sur des synéchies sur 360° et le reste de l'examen est inaccessible.

**Figure 1:**
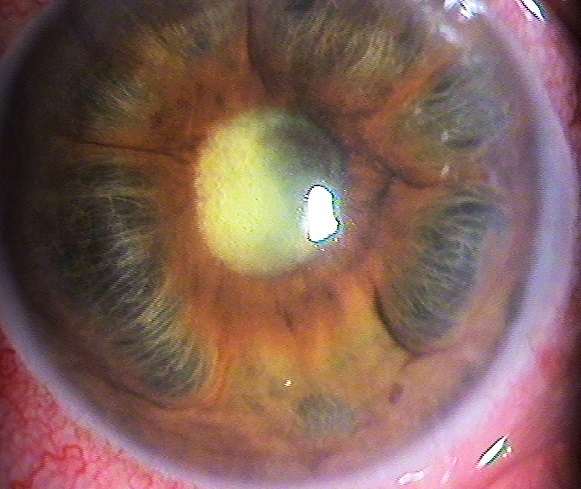
Aspect d'iris tomate sur des synéchies sur 360°

